# A Case of Acute Liver Failure Secondary to Thyrotoxicosis

**DOI:** 10.7759/cureus.115955

**Published:** 2026-09-08

**Authors:** Rakendu Jayasree Rajendran, Abdallah Hussein

**Affiliations:** 1 Internal Medicine, Virtua Health, Camden, USA; 2 Internal Medicine, Virtua Our Lady of Lourdes Hospital, Camden, USA

**Keywords:** acute liver failure, graves' disease, hyperthyroidism, methimazole, thyroid storm, thyrotoxicosis

## Abstract

Thyroid storm is a rare, life-threatening endocrine emergency that can cause multiorgan dysfunction, although acute liver failure (ALF) remains an uncommon manifestation.

A 52-year-old woman with Graves’ disease who had discontinued methimazole presented with three days of nausea, vomiting, diarrhea, melena, generalized weakness, and chest pain. She was lethargic and tachycardic, with a diffusely enlarged thyroid gland, warm skin, bilateral hand tremors, and hypoglycemia. Laboratory testing demonstrated severe thyrotoxicosis, marked hepatocellular injury with an aspartate aminotransferase concentration of 1,059 units per liter (U/L) and an alanine aminotransferase concentration of 955 U/L, and an international normalized ratio of 7.8. Echocardiography showed a left ventricular ejection fraction of 25%-30%, while coronary angiography was normal, and evaluation for viral, autoimmune, and structural hepatobiliary disease was unrevealing.

Treatment with hydrocortisone, propranolol, and methimazole resulted in clinical improvement and substantial biochemical recovery, with normalization of the hepatic and thyroid biochemical parameters at two-month follow-up.

This case emphasizes the importance of considering thyroid storm in patients with ALF and promptly evaluating both thyroid-mediated and hemodynamic contributors to hepatic injury.

## Introduction

Thyroid storm is an uncommon but potentially fatal endocrine emergency with an estimated annual incidence of 0.57-0.76 per 100,000 in the general population and 4.8-5.6 per 100,000 in hospitalized patients [1]. Notably, the extent of circulating thyroid hormone elevation and thyroid-stimulating hormone (TSH) suppression in thyroid storm does not generally exceed what is seen in uncomplicated cases of thyrotoxicosis [1-4]. These patients can present with tachycardia, heart failure, marked fever, agitation, anxiety, delirium, stupor progressing to coma, and gastrointestinal disturbances; acute liver failure (ALF) is a less common but recognized complication [5,6]. Hepatic dysfunction in severe thyrotoxicosis may result from direct thyroid hormone-mediated hepatocyte injury and impaired bilirubin metabolism. It may be further exacerbated by hepatic congestion secondary to high-output or right-sided heart failure [7]. Known triggers for thyroid storm include thyroid and non-thyroidal surgical procedures, trauma, infection, sudden iodine loading (such as from amiodarone therapy), childbirth, and inconsistent adherence to antithyroid medications. When ALF develops, it can be complicated by cerebral edema, cardiac arrhythmias, pulmonary edema, disseminated intravascular coagulation, and hypoglycemia [8].

In this report, we describe a case of ALF arising in the setting of thyroid storm, outlining its diagnostic workup, treatment course, and outcome.

## Case presentation

A 52-year-old woman with a history of acinic cell carcinoma, Graves' disease, prediabetes, and asthma presented to the Emergency Department with a three-day history of nausea, vomiting, diarrhea, and melena. She had completed radiotherapy for acinic cell carcinoma of the parotid gland two weeks before presentation and had been prescribed methimazole 30 milligrams (mg) daily for Graves' disease. According to a family member, she had self-discontinued methimazole after completing radiotherapy. She additionally reported severe generalized weakness and chest pain.

On physical examination, the patient appeared lethargic and had a diffusely enlarged, non-tender thyroid gland with diffusely warm skin. Abdominal examination revealed a soft, non-distended, and non-tender abdomen. She was easily arousable but contributed minimally to the history and appeared lethargic on examination. Fine tremors were present in both hands. She was tachycardic, with a heart rate of 119 beats per minute. She was afebrile with normal blood pressure. The calculated Burch-Wartofsky Point Scale score was 40, not meeting the criteria for thyroid storm [9].

Initial laboratory evaluation (detailed in Table 1) demonstrated hypoglycemia with a random blood glucose of 55 milligrams per deciliter (mg/dL) (reference range: 70-99 mg/dL). She was found to have severe thyrotoxicosis and acute liver injury. Aspartate aminotransferase (AST) was 1,059 units per liter (U/L) (reference range: 10-40 U/L), alanine aminotransferase (ALT) was 955 U/L (reference range: 7-56 U/L), and alkaline phosphatase (ALP) was 86 U/L (reference range: 44-147 U/L). Total bilirubin was 3.2 mg/dL (reference range: 0.2-1.2 mg/dL), including a direct bilirubin of 2.2 mg/dL (reference range: 0.0-0.3 mg/dL). Coagulation studies demonstrated a prolonged prothrombin time (PT) of 86.9 seconds (reference range: 9.0-12.6 seconds), activated partial thromboplastin time (aPTT) of 70.3 seconds (reference range: 25-38 seconds), and an international normalized ratio (INR) of 7.8 (reference range: 0.8-1.1). Serum lactate was elevated at 6.0 millimoles per liter (mmol/L) (reference range: 0.5-2.2 mmol/L), and platelet count was 101 × 10³ per microliter (/µL) (reference range: 150-450 × 10³/µL). Thyroid function tests demonstrated a markedly suppressed TSH level of less than 0.005 milli-international units per liter (mIU/L) (reference range: 0.4-4.5 mIU/L), free thyroxine (free T4) >6 nanogram per deciliter (ng/dL) (reference range: 0.6-1.8 ng/dL), and free triiodothyronine (free T3) >24 picogram per milliliter (pg/mL) (reference range: 2.0-4.4 pg/mL).

**Table 1:** Changes in laboratory parameters from admission to discharge From admission to discharge, AST, ALT, INR, free T4, and free T3 decreased substantially. Total and direct bilirubin remained elevated and were higher at discharge. The INR is reported as a ratio and has no physical unit. The less-than and greater-than symbols indicate values reported below or above the stated measurement threshold. Abbreviations and units: AST, aspartate aminotransferase; ALT, alanine aminotransferase; INR, international normalized ratio; TSH, thyroid-stimulating hormone; T4, thyroxine; T3, triiodothyronine; U/L, units per liter; mg/dL, milligrams per deciliter; ng/dL, nanograms per deciliter; pg/mL, picograms per milliliter; and mIU/L, milli-international units per liter

Laboratory test	Unit	Reference range	Admission	Discharge
AST	U/L	10-40 U/L	1,059	228
ALT	U/L	7-56 U/L	955	358
Total bilirubin	mg/dL	0.2-1.2 mg/dL	3.2	4.5
Direct bilirubin	mg/dL	0.0-0.3 mg/dL	2.2	3.2
INR	Ratio	0.8-1.1	7.8	1.2
Free T4	ng/dL	0.6-1.8 ng/dL	>6.0	1.9
Free T3	pg/mL	2.0-4.4 pg/mL	>24	3.5
TSH	mIU/L	0.4-4.5 mIU/L	<0.005	<0.005

She was admitted and treated with hydrocortisone, propranolol, and methimazole. Further evaluation helped narrow the differential diagnosis. Hepatitis A, B, and C serologies returned negative, as was the autoimmune hepatitis panel. Abdominal ultrasonography demonstrated gallbladder polyps, while computed tomography of the abdomen and pelvis with intravenous and oral contrast was otherwise unremarkable. High-sensitivity cardiac troponin peaked at 59 ng/L (reference range: <14 ng/L). Electrocardiography performed in the Emergency Department demonstrated sinus tachycardia.

Transthoracic echocardiography revealed a severely reduced left ventricular ejection fraction of 25%-30% without regional wall motion abnormalities. Tachycardia-induced cardiomyopathy was diagnosed after left heart catheterization demonstrated angiographically normal coronary arteries. During hospitalization, her symptoms gradually resolved, accompanied by marked biochemical improvement. At discharge, AST had decreased to 228 U/L, ALT to 358 U/L, total bilirubin was 4.5 mg/dL, direct bilirubin was 3.2 mg/dL, free T4 was 1.9 ng/dL, free T3 was 3.5 pg/mL, and INR was 1.2. Upon treatment, her mental status improved back to her baseline, and symptoms fully resolved. She was discharged on metoprolol succinate, losartan, methimazole, and furosemide, with outpatient follow-up arranged with hepatology, endocrinology, and cardiology. Two months later, liver enzymes, bilirubin, free T3, and free T4 had normalized, although TSH remained suppressed, as outlined in Table 1.

## Discussion

Mild elevations in liver function tests occur in a substantial proportion of patients with untreated hyperthyroidism, with reported rates ranging from 15% to 76%; however, frank progression to ALF is rare. Reports specifically documenting ALF associated with thyroid storm are sparse [10-14]. What drives hepatic injury in this setting has not been definitively established, though both direct and indirect pathways have been proposed. Because the liver serves as the principal site of thyroid hormone metabolism, sustained exposure to markedly elevated free thyroid hormone levels, compounded by free-radical production due to the accompanying hypermetabolic state, may cause hepatocyte injury via anoxic injury. Thyroid hormones also affect bilirubin handling by modulating glucuronyl transferase activity and expression of ligandin, a key organic anion-binding protein. Proposed indirect pathways include congestive hepatopathy secondary to thyrotoxicosis-induced right heart failure, hepatotoxicity from methimazole, and autoimmune hepatitis, the latter occurring more frequently in patients with Graves' disease [15,16].

In this patient, methimazole was an improbable cause of the ALF given that she had not been taking it. Moreover, symptomatic liver enzyme elevations linked to methimazole use generally occur within the first three months of high-dose induction treatment, making drug-induced liver enzyme elevation from this agent highly unlikely in her case. Her autoimmune workup was also negative. She was euvolemic to hypovolemic, attributable to her vomiting and diarrhea; hence, congestive hepatopathy is unlikely to be causing the liver injury. Taken together, these findings point toward direct thyroid hormone-mediated hepatocellular injury as the most likely mechanism - a conclusion further supported by the substantial clinical and laboratory improvement that followed methimazole initiation.

Another possible etiology is a coexisting separate autoimmune liver pathology. As many as 10% of patients with Graves' disease harbor a second autoimmune condition, with recognized links to primary biliary cirrhosis and autoimmune hepatitis. The reverse association also holds: Graves' disease is a frequently encountered comorbidity among patients with various chronic liver diseases. Given this bidirectional relationship, patients who present with both thyroid storm and ALF warrant screening for coexisting autoimmune liver disease [17,18]. The mechanisms are described in Figure 1. Standard treatment for thyroid storm combines several strategies: beta-blockade to counter adrenergic overactivity, thionamide therapy (propylthiouracil (PTU) or methimazole) to halt new hormone synthesis, iodinated contrast agents to limit peripheral conversion of T4 to T3, and corticosteroids, which both further reduce T4-to-T3 conversion and address possible concurrent adrenal insufficiency. While PTU has historically been the preferred acute-phase agent owing to its added benefit of blocking peripheral T4-to-T3 conversion, methimazole becomes the preferred choice when liver failure is present, since PTU carries a greater hepatotoxic risk.

**Figure 1:**
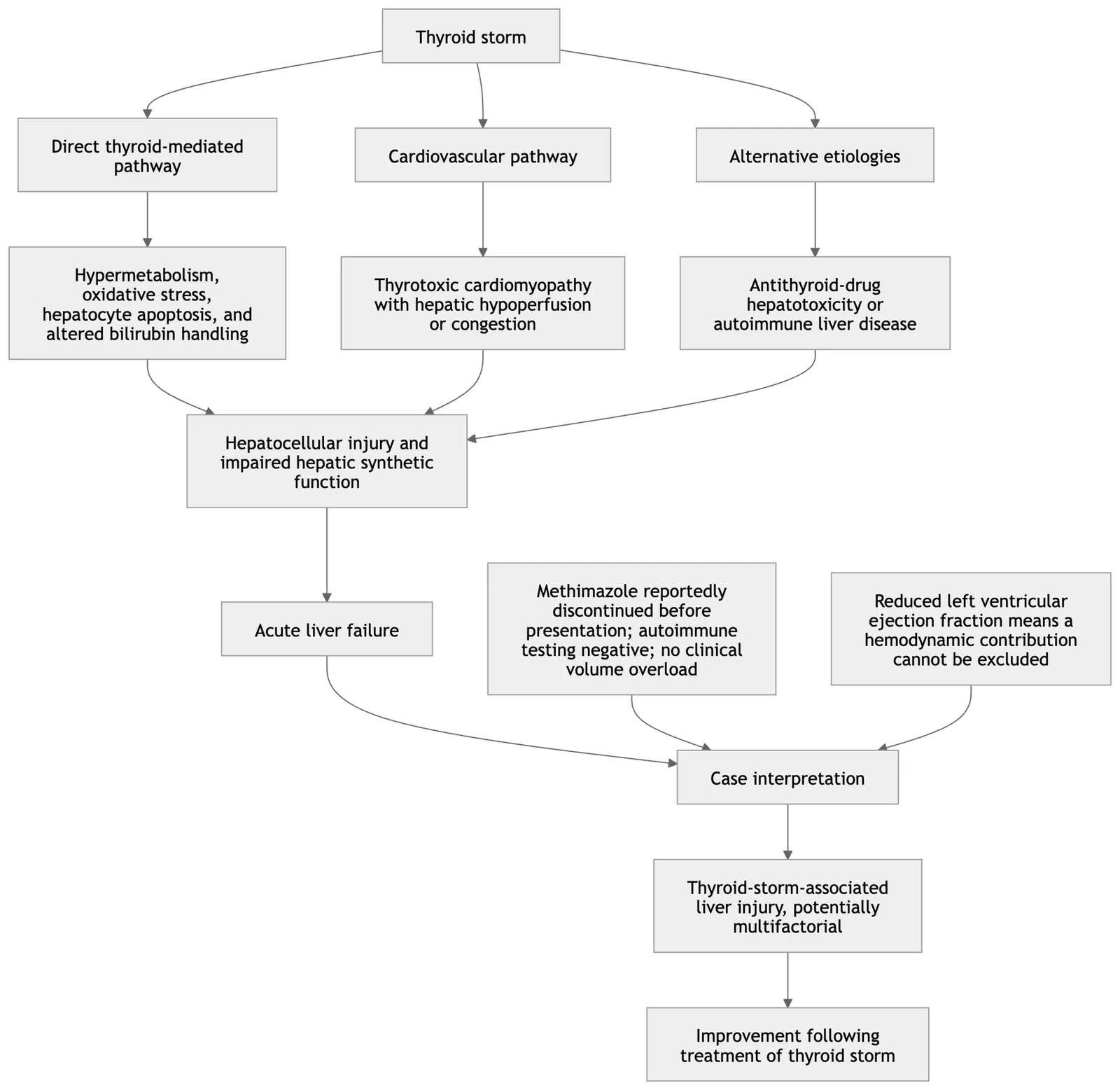
Proposed mechanisms of acute liver failure in thyroid storm and their application to the present case

Although this patient improved substantially with medical management alone, prompt recognition of thyroid storm and early consideration of liver transplantation when clinically warranted remain critical for patients who develop ALF [19].

## Conclusions

ALF is a rare but potentially fatal manifestation of severe thyrotoxicosis and may occur even in the absence of thyroid storm. This case demonstrates that thyroid-related hepatic injury should remain an important consideration in otherwise unexplained ALF, particularly in patients with known thyroid disease, even when classic features of thyroid storm are absent and the Burch-Wartofsky score is below the diagnostic threshold. Prompt recognition and individualized treatment with corticosteroids, beta-blockade, and antithyroid therapy may reverse liver failure and result in favorable clinical outcomes.

## References

[1] Galindo RJ, Hurtado CR, Pasquel FJ, García Tome R, Peng L, Umpierrez GE (2019). National trends in incidence, mortality, and clinical outcomes of patients hospitalized for thyrotoxicosis with and without thyroid storm in the United States, 2004-2013. Thyroid.

[2] Akamizu T (2018). Thyroid storm: a Japanese perspective. Thyroid.

[3] Swee du S, Chng CL, Lim A (2015). Clinical characteristics and outcome of thyroid storm: a case series and review of neuropsychiatric derangements in thyrotoxicosis. Endocr Pract.

[4] Akamizu T, Satoh T, Isozaki O (2012). Diagnostic criteria, clinical features, and incidence of thyroid storm based on nationwide surveys. Thyroid.

[5] Angell TE, Lechner MG, Nguyen CT, Salvato VL, Nicoloff JT, LoPresti JS (2015). Clinical features and hospital outcomes in thyroid storm: a retrospective cohort study. J Clin Endocrinol Metab.

[6] Ngo SY, Chew HC (2007). When the storm passes unnoticed--a case series of thyroid storm. Resuscitation.

[7] Fatima S, Puri R, Patnaik S, Mora J (2024). When a toxic thyroid makes the liver toxic: a case of thyroid storm complicated by acute liver failure. Summer.

[8] Lee WM, Stravitz RT, Larson AM (2012). Introduction to the revised American Association for the Study of Liver Diseases position paper on acute liver failure 2011. Hepatology.

[9] Burch HB (2025). The point scale for thyroid storm - 32 years and (still) counting. Thyroid.

[10] European Association for the Study of the Liver (2017). EASL 2017 Clinical Practice Guidelines on the management of hepatitis B virus infection. J Hepatol.

[11] de Campos Mazo DF, de Vasconcelos GB, Pereira MA, de Mello ES, Bacchella T, Carrilho FJ, Cançado EL (2013). Clinical spectrum and therapeutic approach to hepatocellular injury in patients with hyperthyroidism. Clin Exp Gastroenterol.

[12] Soleimanpour SA (2015). Fulminant liver failure associated with delayed identification of thyroid storm due to heterophile antibodies. Clin Diabetes Endocrinol.

[13] Khemichian S, Fong TL (2011). Hepatic dysfunction in hyperthyroidism. Gastroenterol Hepatol (N Y).

[14] Tanaka Y, Uchida T, Yamaguchi H, Kudo Y, Yonekawa T, Nakazato M (2019). Fulminant hepatitis and elevated levels of sIL-2R in thyroid storm. Endocrinol Diabetes Metab Case Rep.

[15] Yorke E (2024). Co-morbid hypothyroidism and liver dysfunction: a review. Clin Med Insights Endocrinol Diabetes.

[16] Myers RP, Cerini R, Sayegh R, Moreau R, Degott C, Lebrec D, Lee SS (2003). Cardiac hepatopathy: clinical, hemodynamic, and histologic characteristics and correlations. Hepatology.

[17] Boelaert K, Newby PR, Simmonds MJ (2010). Prevalence and relative risk of other autoimmune diseases in subjects with autoimmune thyroid disease. Am J Med.

[18] Czaja AJ, Carpenter HA, Santrach PJ, Moore SB (1993). Genetic predispositions for the immunological features of chronic active hepatitis. Hepatology.

[19] Olivo R, Guarrera JV, Pyrsopoulos NT (2018). Liver transplantation for acute liver failure. Clin Liver Dis.

